# Supplementary material for: Inducing programmed cell death trough MazEF system to combat Staphylococcus aureus: A non-antibiotic treatment candidate
Source: PLoS One. 2024 Dec 5;19(12):e0314867. doi: 10.1371/journal.pone.0314867 (PMC11620692; doi:10.1371/journal.pone.0314867)
Supplement: Attachment — Submitted filename: Response to Reviewers.doc [file pone.0314867.s002.doc]

**Response to Reviewers’ Comments**

**Manuscript Number:** PONE-D-24-30735

**Title: “Inducing programmed cell death trough MazEF system to combat *Staphylococcus aureus*: a non-antibiotic treatment candidate”**

**Dear Dr. Hideo Kato**

**Academic Editor**

**PLOS ONE**

We highly appreciate the editor and anonymous reviewers for their constructive comments which helped us to improve the manuscript. We do appreciate the opportunity to respond to the comments by the journal honorable reviewers.

As suggested, we have revised the manuscript and responded to the reviewers’ comments. Moreover, we have addressed all issues raised by the dear Academic Editor. In this letter, I will briefly summarize the important changes we have made in the manuscript and also respond to the reviewer’s comments.

**Respectfully,**

**Mohammad Hossein Ahmadi**

**Corresponding Author**

**Reviewer #1:** The manuscript is interesting and scientifically sounding. The introduction is clear and comprehensive, the methods are extremely detailed, allowing the replication of the performed experiments, and report a good statistical analysis. The results are well commented in the discussion and the main objective of the study. Still, there are some concerns that need to be addressed:
1. The induction of programmed cell death trough MazEF system to combat Staphylococcus aureus is not currently an option but it may be a candidate. Thus, I suggest the authors to replace "option" with "candidate" in the title of the manuscript and correct the title as: "Inducing programmed cell death trough MazEF system to combat Staphylococcus aureus: a non-antibiotic treatment candidate".

**Our comments:** Very thanks for your good comments. As suggested, we have replaced "option" with "candidate" in the title of the manuscript and corrected the title as: "Inducing programmed cell death trough MazEF system to combat *Staphylococcus aureus*: a non-antibiotic treatment candidate".

2. Please also replace "option" with "candidate" throughout the manuscript text, where applicable.

**Our comments:** As suggested, we have replaced "option" with "candidate" throughout the manuscript text, where applicable.

3. Why the authors used M9 minimal medium with 1% glucose to extract the EDF from E. coli?

**Our comments:** As mentioned in the referenced articles, under the “minimal growth condition”, the production of EDF increases and PCD begins. Therefore, the M9 medium has been used to create such conditions in various articles, and we also used this environment in this study.

4. Why the only EDF or sub-MIC rifampin alone didn’t have significant effect on the CFUs of MRSA or MSSA in your study?

**Our comments:** The MazEF-mediated cell death could be induced by stressful conditions such as the exposure to the rifampin, moreover, the mazEF-mediated cell death is a population phenomenon requiring a quorum-sensing factor called extracellular death factor (EDF). So, it seems that both rifampin and EDF have synergistic effect together.

5. In this study., the supernatant of *E. coli* 25922 was extracted in LB broth (24h and 72h), M9 without glucose, and M9 without amino acids. The authors declare that these supernatants didn’t decrease the CFUs of MRSA or MSSA in comparison to the supernatant of the mid-logarithmic growth phase. What could be the reason?

**Our comments:** As mentioned, the EDF as a quorum-sensing factor is produced in stressful conditions. One of the important stressful conditions could be the starvation. It seems that the most production of EDF is only in M9 medium with amino acids as a minimal medium.

**Reviewer #2:** The interesting topic, the study has well disigned, well performed and the manuscript well written. Due to the increasing resistance rate to conventional thraprutic method and antibiotics, this is very important to seek for novel treatments and new alternatives for the antibiotics.

**Our comments:** Very thanks for your comments.

**Reviewer #3:** The utility of conventional antibiotics for the treatment of bacterial infections has become increasingly strained due to increased rates of resistance coupled with reduced rates of development of new agents. As a result, multidrug-resistant, extensively drug-resistant, and pan-drug-resistant bacterial strains are now frequently encountered.
Alternatives therapeutic approaches to conventional antibiotics need to be explored to ensure that a robust pipeline of effective therapies is available to clinicians. This study introduces a novel non-antibiotic solution to manage invasive bacterial infections.

**Our comments:** Very thanks for your comments.

**Reviewer #4:** Thank you for giving me the opportunity to review this article. In my opinion, the article is scientifically strong, it studies an important topic, it has a good design, and it is well executed. I think this article is suitable for the journal.

**Our comments:** Very thanks for your comments.
